# Comment on Reese et al. 2023 “Vegan Diets from an Allergy Point of View – Position Paper of the DGAKI Working Group on Food Allergy”. Allergologie Select. 2023; 7: 57-83. 

**DOI:** 10.5414/ALX02572E

**Published:** 2025-03-06

**Authors:** Christian Koeder

**Affiliations:** Institute for Prevention and Cancer Epidemiology (IPE), Faculty of Medicine, University of Freiburg, Freiburg, Germany

## Abstract

None.

Sir, – The DGAKI Working Group on Food Allergy recently published a position paper on “Vegan Diets from an Allergy Point of View” [1] in which they present a very helpful overview of several plant foods in relation to potential allergens and potential allergic reactions. For the sake of brevity, the following commentary is plain-spoken. 

Dairy alternatives: The authors state that the use of plant-based drinks (e.g., soya milk, oat milk, etc.) “in the first years of life carries a high risk for nutrient deficiencies” [1]. This statement may be misunderstood. In fact, there are “milk alternatives” specifically formulated for young children, and it is unclear how the inclusion of moderate amounts of regular soya drinks (fortified with vitamin B12 and calcium) would necessarily increase the risk of nutrient deficiencies. In complementary feeding, soya drinks (without added sugar) can be used in small quantities, just like cow’s milk should only be used in small quantities. 

Vitamin B12: The authors state that “vitamin B12 […] must be substituted daily” [1]. This is not necessarily the case. Decades of experience with completely vegan diets clearly indicate the adequacy of weekly oral supplementation of vitamin B12 with ~ 2,000 – 2,500 µg once per week (non-pregnant adults) [2, 3], although 500 µg at least 3 times a week have also been suggested [4]. 

Eicosapentaenoic acid (EPA) and docosahexaenoic acid (DHA): The authors state that, in vegans, a “sufficient intake of [...] the long-chain omega-3 fatty acids EPA and DHA from marine microalgae […] must be ensured” [1]. While this is a widely given recommendation [4], its true clinical benefit awaits confirmation [4, 5, 6, 7, 8], especially for vegans with a high intake (e.g., 4 g/d) of α-linolenic acid and a high intake of other foods (including spices) with anti-inflammatory and anti-thrombotic properties [2]. 

Calcium: The authors state that “on account of the poorer availability of […] calcium, […] in a purely plant-based diet, the daily requirements are increased” [1]. However, while calcium bioavailability from spinach and Swiss chard appears to be low, that from bok choy, kale, and Napa cabbage appears to be higher than and that from calcium-fortified drinks and calcium-rich water similar to that from cow’s milk [2]. Nevertheless, more human bioavailability studies are needed. 

VeChi Diet Study: The authors state that the VeChi Diet Study showed that in vegan children, the requirement of vitamin B12 is “only met to a limited extent” [1]. However, the VeChi Diet study showed that, among the 139 vegan children (age: 1 – 3 years), the median (25 – 75^th^ percentile) vitamin B12 intake (including supplements) was ~ 71 (6 – 334) µg/d, compared to 1.6 (1.1 – 2.6) µg/d in omnivorous children [9]. 

Allergy risk and dietary diversity: The authors state that “a vegan diet […] contradicts the recommendation for a varied diet consisting of as many food groups as possible [...].” What comprises a food group is a matter of opinion, and vegan diets can be as varied (by number of different foods) as omnivorous diets. Intestinal microbiome diversity does not appear to be lower in vegans [10]. 

Nutrient profile of vegan diets: The authors state: “[...] vegan means that many nutrients are missing or not available in sufficient quantities.” [1] This statement is contradicted by vegan sources of key nutrients and by vegans with adequate nutrient status [2]. 

Nutrition knowledge required: The authors state: “To ensure an adequate supply of vitamin B12, but also of calcium, iron, iodine, zinc, as well as high-quality protein and long-chain omega-3 fatty acids (EPA/DHA), an in-depth study of the subject of nutrition, time investment, and supplementation, with regard to various nutrients, is required.” [1] However, vegans can cover their needs for key nutrients with a multivitamin supplement, and a good source of calcium and omega-3 fatty acids [2]. 

V-label: The authors state that vegan labels should fulfil the role of informing those with allergies to animal products. However, this has never been the intended purpose of vegan labels. Vegan labels are meant to help those who wish to buy vegan products. Most common vegan labels allow for traces of animal products, but the “Plamil vegan logo” does not [11]. 

Vegan diets for infants: The authors state that “[a] vegan diet is particularly critical in infants and toddlers, as the required quantities needed to ensure adequate supply often exceed the capacity of (young) children.” [1] Decades of experience and current evidence (which is limited) do no confirm this statement [8, 9, 12, 13, 14]. 

## Funding 

None. 

## Conflict of interest 

None. C.K. is the author of several books on the topic of vegan nutrition and veganism (from which he does not earn any relevant income). 

## References

[1] Reese I Schäfer C Ballmer-Weber B Beyer K Dölle-Bierke S van Dullemen S Jappe U Müller S Schnadt S Treudler R Worm M Vegan diets from an allergy point of view - Position paper of the DGAKI working group on food allergy. Allergol Select. 2023; 7: 57–83. 37056444 10.5414/ALX02400E PMC10088878

[2] Koeder C Perez-Cueto FJA Vegan nutrition: a preliminary guide for health professionals. Crit Rev Food Sci Nutr. 2022; 64: 1–38. 35959711 10.1080/10408398.2022.2107997

[3] Walsh S Plant Based Nutrition and Health. St Leonards-on-Sea: Vegan Society; 2003.

[4] Craig WJ Mangels AR Fresán U Marsh K Miles FL Saunders AV Haddad EH Heskey CE Johnston P Larson-Meyer E Orlich M The Safe and Effective Use of Plant-Based Diets with Guidelines for Health Professionals. Nutrients. 2021; 13: 4144. 34836399 10.3390/nu13114144 PMC8623061

[5] Burdge GC α-linolenic acid interconversion is sufficient as a source of longer chain ω-3 polyunsaturated fatty acids in humans: An opinion. Lipids. 2022; 57: 267–287. 35908848 10.1002/lipd.12355

[6] Abdelhamid AS Brown TJ Brainard JS Biswas P Thorpe GC Moore HJ Deane KH Summerbell CD Worthington HV Song F Hooper L Omega-3 fatty acids for the primary and secondary prevention of cardiovascular disease. Cochrane Database Syst Rev. 2020; 3: CD003177. 32114706 10.1002/14651858.CD003177.pub5 PMC7049091

[7] Agnoli C Baroni L Bertini I Ciappellano S Fabbri A Papa M Pellegrini N Sbarbati R Scarino ML Siani V Sieri S Position paper on vegetarian diets from the working group of the Italian Society of Human Nutrition. Nutr Metab Cardiovasc Dis. 2017; 27: 1037–1052. 29174030 10.1016/j.numecd.2017.10.020

[8] Melina V Craig W Levin S Position of the Academy of Nutrition and Dietetics: Vegetarian Diets. J Acad Nutr Diet. 2016; 116: 1970–1980. 27886704 10.1016/j.jand.2016.09.025

[9] Weder S Keller M Fischer M Becker K Alexy U Intake of micronutrients and fatty acids of vegetarian, vegan, and omnivorous children (1 – 3 years) in Germany (VeChi Diet Study). Eur J Nutr. 2022; 61: 1507–1520. 34855006 10.1007/s00394-021-02753-3 PMC8921058

[10] Ferrocino I Di Cagno R De Angelis M Turroni S Vannini L Bancalari E Rantsiou K Cardinali G Neviani E Cocolin L Fecal Microbiota in Healthy Subjects Following Omnivore, Vegetarian and Vegan Diets: Culturable Populations and rRNA DGGE Profiling. PLoS One. 2015; 10: 10.1371/journal.pone.0128669 PMC4452701 26035837

[11] Plamil Foods. 2024; ‘Vegan Trademark’, https://plamilfoods.co.uk/vegan-2/

[12] Mangels AR Messina V Considerations in planning vegan diets: infants. J Am Diet Assoc. 2001; 101: 670–677. 11424546 10.1016/S0002-8223(01)00169-9

[13] Moilanen BC Vegan diets in infants, children, and adolescents. Pediatr Rev. 2004; 25: 174–176. 15121909 10.1542/pir.25-5-174

[14] Hay G Fadnes L Meltzer HM Arnesen EK Henriksen C Follow-up of pregnant women, breastfeeding mothers and infants on a vegetarian or vegan diet. Tidsskr Nor Laegeforen. 2022; 142: 10.4045/tidsskr.21.0847 35510463

